# Editorial: Methods and applications of diffusion MRI tractometry

**DOI:** 10.3389/fnins.2025.1737026

**Published:** 2025-11-19

**Authors:** Bramsh Qamar Chandio, Kurt G. Schilling, Julio E. Villalón-Reina

**Affiliations:** 1 Department of Chemical and Biomedical Engineering, Statler College of Engineering and Mineral Resources, West Virginia University, Morgantown, WV, United States; 2 Imaging Genetics Center, Stevens Neuroimaging and Informatics Institute, Keck School of Medicine, University of Southern California, Marina del Rey, CA, United States; 3 Department of Radiology and Radiological Sciences (VUMC), Vanderbilt University Medical Center, Nashville, TN, United States

**Keywords:** diffusion MRI (dMRI), tractography, tractometry, white matter (WM) microstructural organization, connectome

Advances in diffusion MRI (dMRI) have enabled increasingly precise reconstruction and quantification of white matter pathways, and tractometry has emerged as a powerful framework for examining microstructural and macrostructural properties *along* the length of specific tracts. The concept of tractometry arose from early efforts to quantify dMRI metrics along white matter pathways rather than across broad regions. Early implementations summarized each bundle with a single averaged dMRI measure (e.g., mean fractional anisotropy), representing a pioneering and foundational step in combining tractography with diffusion tensor imaging (DTI). While this approach laid important groundwork for tract-specific analyses, it inherently overlooked spatial heterogeneity along the tract. The field advanced with the introduction of the Pointwise Assessment of Streamline Tractography Attributes (PASTA) introduced by Jones et al. (2005), which enabled pointwise sampling of microstructural measures along the tract length and established the foundation for along-tract analyses. The term *tractometry* was first formally introduced by Bells et al. (2011), defining it as a multimodal framework for quantitative assessment of white matter microstructure along specific tracts. Modern tractometry approaches now integrate diffusion- and microstructure-based metrics such as DTI, neurite orientation dispersion and density imaging (NODDI), and diffusion kurtosis imaging (DKI) to generate spatially detailed along-tract profiles for statistical analysis across individuals and populations, or broadly, any quantitative analysis utilizing streamlines. Collectively, these developments have established tractometry as a powerful framework for mapping white matter alterations with high anatomical specificity.

This Research Topic brings together methodological innovations, translational applications, and large-scale analyses that showcase how tract-specific profiling can advance our understanding of development, disease mechanisms, individual variability, and structure–function relationships. Collectively, the contributions highlight growing advancements in tractometry workflows, the value of open and reproducible tools, and the expanding range of neuroscientific questions that can be addressed using tractometry approaches. This Research Topic features sixteen articles spanning clinical, developmental, computational, and translational domains, illustrating both the breadth and future promise of tractometry.

Bosticardo et al. examined myelin-weighted structural connectivity across the lifespan, demonstrating that tractography-derived myelin measures capture characteristic phases of maturation and degeneration that unfold along specific pathways. Their work illustrates how myelin-sensitive tractometry can complement dMRI-based metrics and enhance our understanding of age-related white-matter trajectories.

Weber et al. used fMRI-guided diffusion MRI tractography to map white matter changes in autism across development. They found early reductions in callosal and periventricular tracts in infants, expanding to widespread disruptions in adolescents and adults. The study highlights edge-density mapping as a sensitive tool for early detection and longitudinal tracking of ASD-related network alterations.

González Rodríguez et al. introduced an open and modular software platform designed to support reproducible tractography, bundle segmentation, clustering, and visualization. Their contribution lowers practical barriers to tractometry adoption and promotes standardized workflows for both research and clinical studies.

Poo et al. presented a simulation framework that generates realistic white-matter streamlines with known ground-truth labels, enabling objective benchmarking of clustering and segmentation methods. This resource directly addresses a major validation challenge in tractometry by providing a controlled environment for algorithm comparison.

Mendoza et al. focused on the superficial white matter and demonstrated that targeted filtering of short U-fibers can substantially improve test–retest reproducibility. By reducing anatomically implausible streamlines, their work shows how thoughtful preprocessing can sharpen effect sizes and enhance sensitivity to group differences.

Kruper et al. leveraged a large population dataset to extract along-tract diffusion profiles, quantify their heritability, and relate tract-specific features to individual traits. By releasing their processed outputs and tools to the community, the authors provide an important normative resource for future tractometry research.

Xue et al. introduced a supervised contrastive learning framework that improves the prediction of cognitive performance from harmonized multisite tractography data. Their findings highlight the synergy between tractometry and modern machine-learning approaches for modeling brain–behavior relationships at scale.

Guberman et al. investigated youth with traumatic brain injury and disruptive behavior, revealing tract-specific microstructural alterations and sex-dependent effects. Their results demonstrate the value of along-tract analyses for uncovering subtle developmental differences that global metrics may overlook.

Yang Z. et al. explored structural connectivity in insular glioma patients, showing that genetic subtype was associated with distinct patterns of white-matter disruption. Their findings illustrate how tract-focused network analysis can illuminate tumor-related reorganization and support more individualized characterization of lesion impact.

Meisler et al. provided a practical guide for integrating functional regions of interest with tractography-defined pathways, enabling the creation of functionally informed sub-bundles. This work facilitates multimodal tractometry pipelines that link white-matter architecture with functional specialization.

Persson and Moreno addressed streamline redundancy in tractography by proposing a framework to estimate and reduce excessive or overlapping streamlines. By improving anatomical specificity and computational efficiency, their method supports more stable and interpretable tract-level metrics.

Yang S. et al. reviewed diffusion-tensor methods in small-vessel disease and highlighted the limitations of traditional voxel-based and region-of-interest (ROI) approaches. They emphasized the need for tract-specific methods that better localize cerebrovascular injury along affected pathways.

Hernandez-Gutierrez et al. evaluated multi-tensor fixel-based metrics and demonstrated improved robustness in crossing-fiber regions, particularly in multiple sclerosis. Their tractometry pipeline showed enhanced sensitivity to lesion-related abnormalities and illustrates the advantages of richer microstructural modeling.

Quizhpilema et al. investigated amyotrophic lateral sclerosis and revealed asymmetric degeneration extending beyond classic motor pathways. Their along-tract analyses reinforce the concept of amyotrophic lateral sclerosis (ALS) as a network-level disorder rather than a purely motor disease.

Behroozi et al. highlighted the role of *ex-vivo* diffusion imaging in large-animal models as a translational bridge between histology and human research. By outlining how *ex-vivo* data can validate microstructural interpretations, their work supports the biological grounding of tractometry measures.

Witt et al. extended tractometry to the spinal cord and showed that profiling diffusion and macrostructural features across cervical levels increases sensitivity to localized pathology in multiple sclerosis. Their findings demonstrate that the along-tract concept can be meaningfully applied beyond the brain.

From this Research Topic, we see that tractometry has evolved into a broad and integrative framework encompassing connectome-wide analyses of both long-range and short association pathways, including functionally defined, non-human, and spinal cord applications. Across the lifespan and in diverse conditions, from cognition and multiple sclerosis to autism, small vessel disease and gliomas, tractometry enables precise mapping of white matter microstructure using diffusion-based, fixel-based, and connectomic measures. Recent advances extend beyond traditional DTI and tract-averaged analyses, to incorporating myelin-sensitive metrics, morphometry, and differential tractography. Together, these developments highlight a growing ecosystem of tractometry tools that bridge structure, function, and pathology across species and systems, advancing whole-brain, circuit-level understanding of the human and non-human connectome.
